# Gastrointestinal Fate and Receptor-Mediated Mechanism of GPSGPQGSR, an Intestinal Barrier-Protective Collagen Peptide from ALASKA Pollock Skin

**DOI:** 10.3390/md24060203

**Published:** 2026-06-08

**Authors:** Qianru Chen, Zheng Zhao, Fengwu Wang, Tiejun Chen, Ting Ding, Jingyuan Li, Zhuang Yao, Yang Deng, Ying Wang

**Affiliations:** 1 College of Food Science and Engineering, Qingdao Agricultural University, Qingdao 266109, China; chenqianru@qau.edu.cn (Q.C.); zhaozheng25@mails.ucas.ac.cn (Z.Z.); dengyang719@hotmail.com (Y.D.); 2 Key Laboratory of Special Food Processing (Co-Construction by Ministry and Province), Ministry of Agriculture Rural Affairs, Qingdao Agricultural University, Qingdao 266109, China; 3 Shandong Technology Innovation Center of Special Food, Qingdao 266109, China; 4 Qingdao Special Food Research Institute, Qingdao 266109, China; 5 Sino-Danish College, University of Chinese Academy of Sciences, Beijing 101408, China

**Keywords:** marine collagen peptide, gastrointestinal fate, mechanism, intestinal barrier function, receptor

## Abstract

Marine-derived collagen peptides exhibit potent intestinal barrier protection; however, their gastrointestinal fate and molecular targets remain unclear, limiting their practical applications. This study investigated the digestive stability and transepithelial transport of GPSGPQGSR, a mucoprotective peptide from Alaska pollock (*Gadus chalcogrammus*) skin, using simulated gastrointestinal digestion, a Caco-2 cell transport model, and an UPLC-QTOF-MS/MS. The results showed that GPSGPQGSR was a digestion-resistant peptide that reached the intestinal epithelium intact. Although brush border membrane enzymes partially hydrolysed the peptide, 42.16% of intact GPSGPQGSR remained in the luminal compartment after 2 h of incubation. No intact peptide was detected in the basolateral compartment. Molecular docking and 100 ns molecular dynamics simulations identified TLR2 (−14.936 kcal/mol) and PAR2 (−10.154 kcal/mol) as high-affinity extracellular targets of GPSGPQGSR, with stable peptide–receptor interactions and extensive hydrogen bonding networks between the peptide and each receptor (RMSD of 1.8 Å and 2.2 Å, respectively). Pharmacological blockade of TLR2 or PAR2 abolished the protective effects of GPSGPQGSR. These findings demonstrate that GPSGPQGSR acts as a digestion-resistant extracellular signalling peptide that reaches the intestinal epithelium intact and protects barrier function through apical TLR2 and PAR2, providing a mechanistic basis for the rational development of marine collagen peptides for improving intestinal health.

## 1. Introduction

The human gastrointestinal (GI) tract is a complex physiological environment where the dynamic interactions between host epithelial cells and diet compositions are crucial for keeping the mucosa barrier integrity and whole-body health [1]. Disruption of this fine balance, marked by alterations in tight junction (TJ) compounds and increased paracellular permeability between cells, therefore plays a pivotal role in the pathogenesis of many metabolism-related, immunity-related and inflammation-related diseases [2,3]. The global burden of intestinal mucosal dysfunction has increased markedly over the past decade, with the worldwide incidence of inflammatory bowel disease (IBD) and associated barrier-related disorders rising substantially across both developed and developing nations, imposing significant socioeconomic burdens on healthcare systems [4,5]. Early nutritional interventions targeting the intestinal barrier have demonstrated considerable promise in preventing health complications associated with barrier impairment [1,5,6]. Therefore, there is an urgent need to identify and develop novel bioactive compounds capable of maintaining homeostasis [7,8].

Among diverse natural sources, bioactive peptides derived from dietary proteins have been extensively studied for their intestinal protective effects [8,9,10,11,12,13]. Compared with synthetic pharmacological agents, marine-derived bioactive peptides offer several compelling advantages, including high biocompatibility, low immunogenicity, structural diversity arising from unique marine biodiversity, and superior safety profiles, making them particularly attractive candidates for functional food and nutraceutical applications [14,15]. Collagen peptides sourced from marine organisms have emerged as particularly promising candidates owing to their excellent biocompatibility, structural stability, and potent tissue repair properties [16,17,18]. Recent robust preclinical and clinical evidence has demonstrated that supplementation with marine fish sidestream-derived protein hydrolysates and collagen peptides can effectively suppress experimental colitis, alleviate symptoms of irritable bowel syndrome (IBS), and improve epithelial barrier dysfunction [19,20,21,22,23,24].

Despite these encouraging macroscopic findings, the underlying molecular mechanisms remain poorly understood. Contemporary peptide therapeutics face a critical bioavailability paradox [25,26]. Traditional pharmacological paradigms often assume that orally administered peptides must be absorbed, permeate the epithelium, and enter systemic circulation to exert biological effects. However, intact large peptides generally exhibit very low membrane permeability and variable stability within the digestive tract [26]. Emerging studies reveal that certain digestion-resistant peptides exert biological effects without cellular internalisation. Instead, they act at the apical membrane surface by dynamically interacting with membrane-bound sensory or immune receptors, such as G-protein-coupled receptors (GPCRs) and Toll-like receptors (TLRs), on the epithelial brush border, thereby initiating intracellular signalling cascades that promote tight junction assembly and tissue repair [27,28,29].

Alaska pollock (*Gadus chalcogrammus*) is one of the most commercially harvested marine fish species globally, generating substantial quantities of skin and bone byproducts during industrial processing. The valorisation of these underutilised side streams through enzymatic hydrolysis represents a sustainable and economically viable strategy for bioactive peptide production [30]. Our previous in vitro and in vivo investigations demonstrated that specific collagen peptides derived from Alaska pollock skin, particularly the highly active sequence GPSGPQGSR, effectively protect against intestinal epithelial barrier dysfunction by enhancing tight junctions and mitigating inflammatory responses [19,20,23,31]. However, the precise gastrointestinal fate and the receptor-level molecular mechanism underlying the barrier-protective activity of this peptide have not yet been systematically characterized, representing a critical knowledge gap that limits its rational development as a functional ingredient.

To address this gap, the present study comprehensively investigated (i) the resistance of GPSGPQGSR to simulated upper gastrointestinal digestion; (ii) its epithelial distribution and transport behaviour across a Caco-2 monolayer model; and (iii) its potential association with apical receptor-mediated protective mechanisms, with candidate targets evaluated through molecular docking, molecular dynamics simulations, and pharmacological inhibition assays. The findings are expected to establish a mechanistic foundation for the development of marine collagen peptides as targeted, luminally acting functional ingredients for intestinal health management.

## 2. Results and Discussion

### 2.1. In Vitro Gastrointestinal Stability of GPSGPQGSR

**Figure 1:**
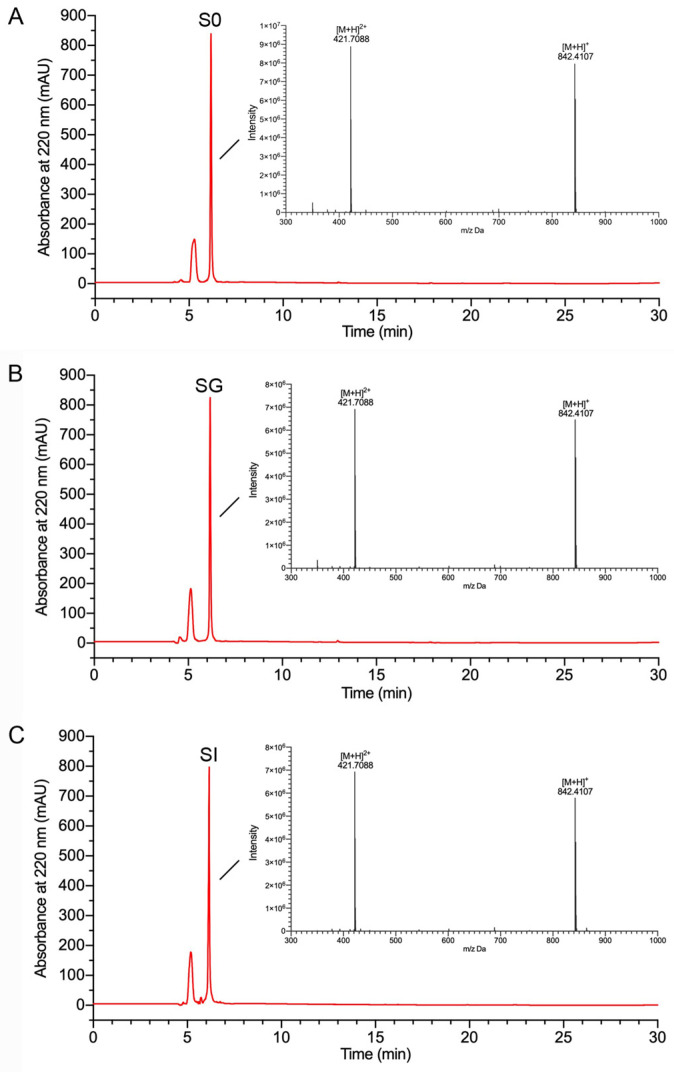
Gastrointestinal stability of GPSGPQGSR. (**A**) GPSGPQGSR before simulates gastrointestinal digestion; (**B**) GPSGPQGSR after simulates gastric digestion; (**C**) GPSGPQGSR after simulates gastrointestinal digestion.

The gastrointestinal stability of the nonapeptide GPSGPQGSR was evaluated using an in vitro static INFOGEST-based digestion model and UPLC-Q-TOF-MS/MS. Figure 1 shows that GPSGPQGSR is highly resistant to gastrointestinal proteolytic degradation. Initially, the nonapeptide displayed a single peak in RP-HPLC at around 6.0 min, with a precise molecular weight of 841.4 Da, confirmed by high-resolution mass spectrometry. After 120 min of gastric digestion with pepsin at pH 2.0, the peak remained unchanged. Even after an additional 150 min of intestinal digestion with pancreatin, the primary peak still dominated the chromatogram, indicating that GPSGPQGSR possesses high digestive stability and can reach the intestinal epithelium as the intact 9-peptide following oral administration.

The exceptional gastrointestinal stability of this peptide can be primarily attributed to its distinctive sequence composition. Proline (Pro)-rich peptides exhibit high stability and resistance to degradation due to their unique structural properties [32]. The incorporation of two Pro residues introduces cyclic pyrrolidine rings, which significantly constrain the backbone angle and create substantial steric hindrance against the active sites of non-specific proteases, such as pepsin [33]. Furthermore, the sequence is enriched with Glycine (Gly, 3 residues), which plays a pivotal role in facilitating the adoption of highly compact spatial conformations by the peptide backbone [34]. Their minimal steric hindrance and associated flexibility allow for dynamic structural adaptations, which are crucial for both the stability and functionality of peptide structures [35]. Although pancreatic trypsin typically cleaves at the carboxyl side of Arginine (Arg) and Lysine (Lys), the single Arg residue in GPSGPQGSR is strategically positioned at the absolute C-terminus. As a result, the internal structural backbone effectively evades cleavage by trypsin. Collectively, these structural attributes confer remarkable stability to GPSGPQGSR, enabling it to resist rapid degradation within the lumen.

### 2.2. Metabolism and Transportation of GPSGPQGSR in Intestinal Epithelial Cells

**Figure 2:**
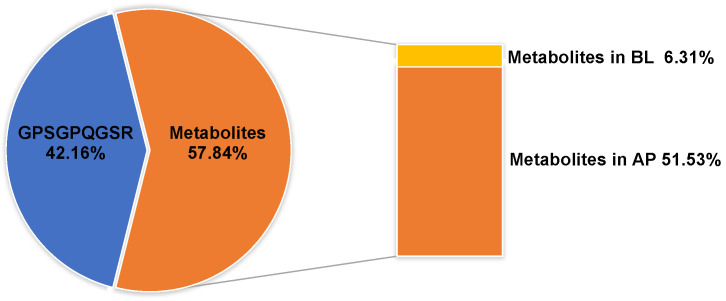
Metabolic fate and distribution of GPSGPQGSR in intestinal epithelial cells.

To study the metabolic fate and distribution of GPSGPQGSR with intestinal epithelium, a Caco-2 cell monolayer model was used. After 2 h, UPLC-Q-TOF-MS/MS analyzed metabolic profiles in the apical (AP) and basolateral (BL) chambers and intracellular lysate. As shown in Figure 2, the overall metabolic rate of GPSGPQGSR by the Caco-2 cells was determined to be approximately 57.84%. Notably, the spatial distribution of these substances was highly polarized. Specifically, 42.16% of the administered dose remained as the intact peptide, and 51.53% existed as metabolic fragments, both of which were entirely retained in the AP chamber. In contrast, the transport rate of metabolites to the BL chamber was exceedingly low, accounting for merely 6.31%, and the cellular absorption was too low to be detected.

**Figure 3:**
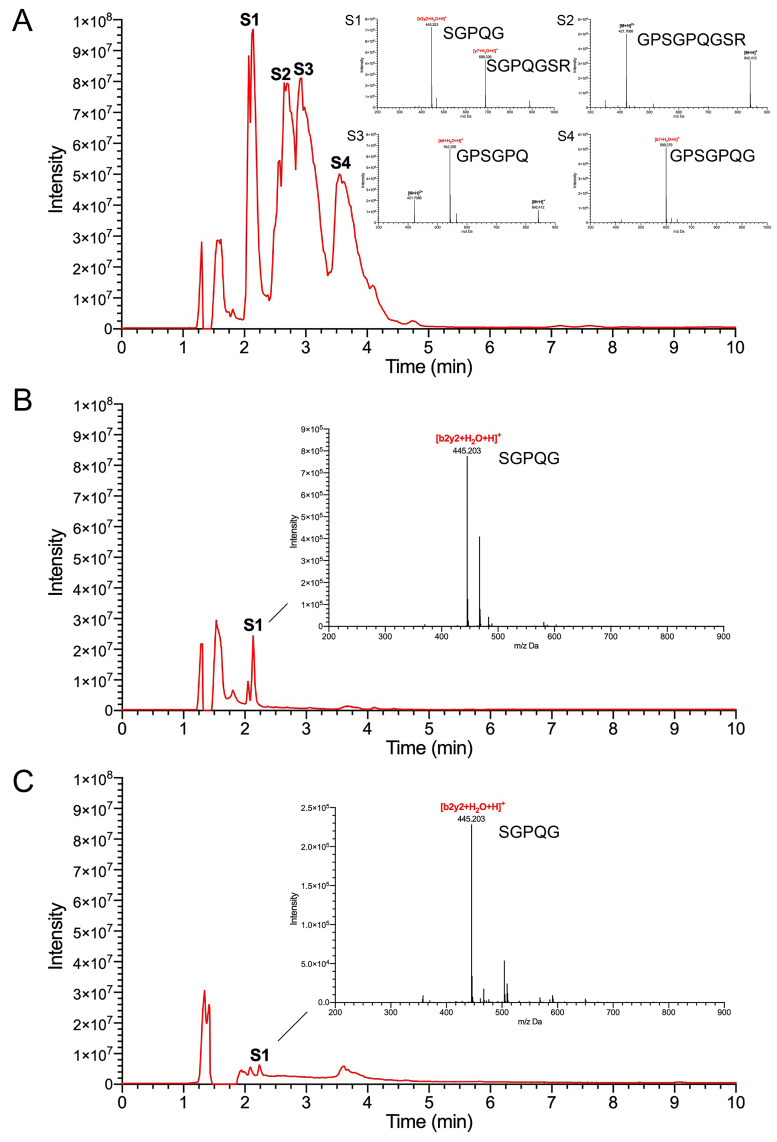
Metabolism and transport of GPSGPQGSR in the Caco-2 cell model. Samples from the AP chamber (**A**), BL chamber (**B**), and intracellular lysates (**C**) after 2 h incubation with GPSGPQGSR.

As illustrated in Figure 3A, the apical (AP) chamber, which represents the intestinal lumen side, exhibited a complex metabolic profile. In addition to the intact nonapeptide GPSGPQGSR (peak S2), four distinct oligopeptide fragments were identified: SGPQG and SGPQGSR (co-eluting in peak S1), GPSGPQ (peak S3), and GPSGPQG (peak S4). This fragmentation pattern indicates that GPSGPQGSR is susceptible to partial enzymatic cleavage by peptidases located on the intestinal brush border membrane. In stark contrast, the metabolic profiles of the intracellular lysate (Figure 3C) and the basolateral (BL) chamber (Figure 3B) showed a complete absence of the intact nonapeptide. Only trace amounts of the pentapeptide metabolite SGPQG (peak S1) were detected, suggesting that only highly specific small fragments are capable of being absorbed or translocated. Given the exceedingly limited intracellular accumulation and basolateral translocation, the majority of the substances (over 93%) remain confined to the luminal compartment. Therefore, it can be strongly inferred that GPSGPQGSR and its metabolites primarily exert their biological functions on the luminal side, acting directly on the surface of the intestinal epithelium.

The transport and metabolic profiles observed are in strong alignment with the well-established physiological constraints of the intestinal barrier. As highlighted by Wang et al. (2018), intact oligopeptides comprising more than four or five amino acids typically demonstrate notably low paracellular and transcellular permeability [26]. The remarkable apical retention (>93%) of GPSGPQGSR and its metabolites provides important theoretical evidence for its mechanism of action. By remaining concentrated within the intestinal lumen and resisting rapid intracellular internalization, GPSGPQGSR is optimally positioned to function as an extracellular ligand. This extensive luminal retention enables the peptide to dynamically and persistently engage with apically localized membrane receptors, thereby initiating downstream intracellular signalling cascades for tight junction repair without necessitating translocation across the epithelial barrier.

### 2.3. Virtual Screening and Molecular Docking Analysis of Specific Extracellular Targets

Based on strong evidence for apical retention, we hypothesized that the bulky nonapeptide GPSGPQGSR may strengthen intestinal barrier integrity by interacting with transmembrane proteins on the luminal surface. To computationally identify candidate extracellular targets, virtual screening and molecular docking were performed. Ten candidate surface proteins with established roles in intestinal barrier regulation were selected as docking targets, including three tight junction proteins (Claudin-1, Claudin-2, and Occludin) and seven membrane-bound receptors (TLR2, PAR2, TGFβR, EGFR, VEGFR, CaSR, and CXCR1). The selection of these candidates was based on their documented involvement in epithelial barrier homeostasis, inflammatory signalling, and tight junction regulation in prior literature, as well as findings from our previous studies [19,23,36,37,38,39].

As depicted in Table 1, the predicted binding affinities of GPSGPQGSR varied considerably across the ten targets. The nonapeptide exhibited remarkably high computational binding affinities for TLR2 (−14.936 kcal/mol) and PAR2 (−10.154 kcal/mol), substantially exceeding those predicted for TGFβR (−7.714 kcal/mol) and EGFR (−7.615 kcal/mol). The critical tight junction structural proteins Occludin (−6.621 kcal/mol) and Claudin-1 (−6.513 kcal/mol) exhibited relatively moderate predicted binding affinities, while the remaining four targets, Claudin-2, VEGFR, CaSR, and CXCR1, showed scores above −6.0 kcal/mol. This pronounced energy disparity in predicted binding energy suggests, from a computational standpoint, that GPSGPQGSR may preferentially engage with TLR2 and PAR2 over structure tight junction proteins such as Occludin and Claudin-1. Rather than acting as a physical intercalator of tight junction complexes, the peptide may function as a potential signalling ligand for membrane-bound receptors, a hypothesis that requires experimental validation.

**Figure 4:**
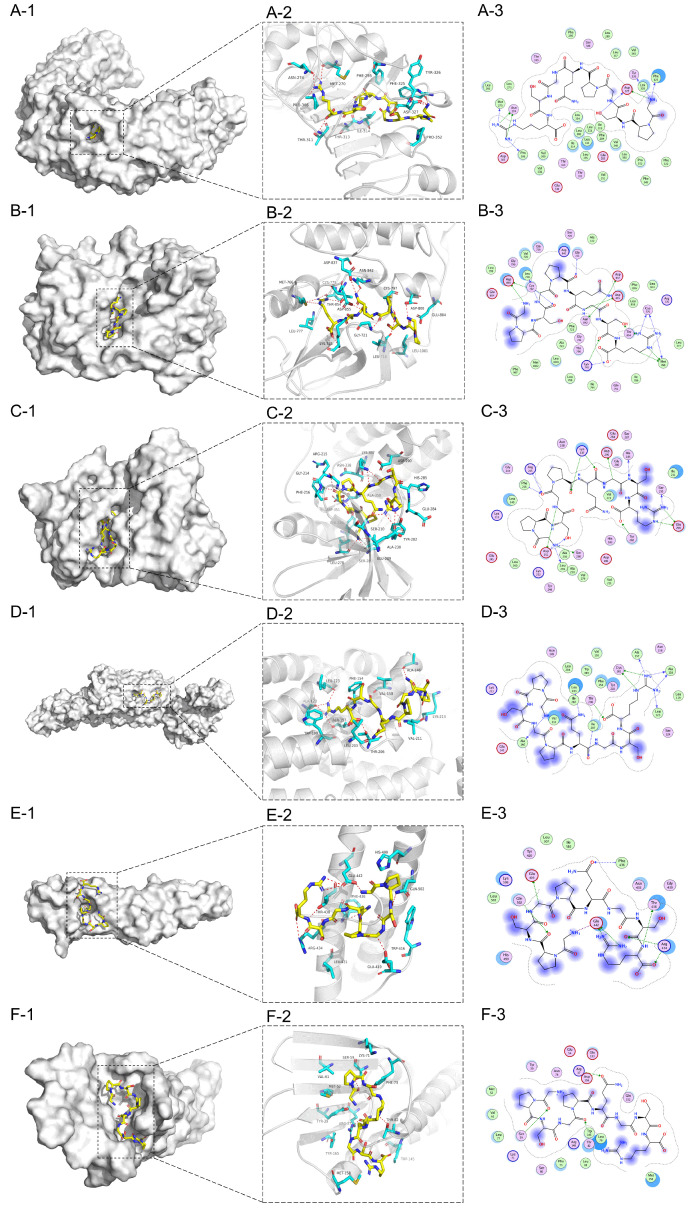
Binding model of the peptide with targets TLR2 (**A**), EGFR (**B**), TGFβR (**C**), PAR2 (**D**), Occludin (**E**) and Claudin-1 (**F**). 1, represents depict surface binding mode, 2, represents three-dimensional binding mode, 3, represents two-dimensional binding mode. In the surface representation, the receptor is displayed in white. In the 3D diagrams, the ligand is colored yellow, the surrounding residues in the binding pocket are shown in cyan, the receptor main chain is represented as a light blue ribbon, and red dashed lines indicate hydrogen bonds. In the 2D diagrams, blue dashed lines indicate hydrogen bonds with the receptor main chain, while green dashed lines denote hydrogen bonds with side chains.

To elucidate the atomic-level interactions potentially underlying these predicted affinities, the top six peptide–receptor complexes were visualized using three-dimensional surface models, three-dimensional binding pocket models, and two-dimensional interaction diagrams (Figure 4).

As primary targets, both TLR2 and PAR2 possess deep and geometrically compatible binding pockets for the nonapeptide. Within the predicted TLR2-GPSGPQGSR complex, the peptide is modeled to form a robust hydrogen bond network with key residues, including Asn62, Ser85, Tyr111, ThrA161, and Asp160, complemented by an attractive charge interaction with Arg63 and extensive van der Waals and hydrophobic interactions (e.g., Pro135, Asn158, Met159, Gly133), effectively anchoring the ligand. Similarly, the predicted PAR-GPSGPQGSR complex is stabilized by multiple hydrogen bonds with Pro231, Arg1154, Gln317, Thr1151 and Ser60, alongside salt bridges with Lys1147 and Glu232. A substantial van der Waals interaction network involving over 15 residues (e.g., Ile216, Leu230, Tyr1139, Phe59) synergistically secures the peptide. These extensive predicted interaction networks are consistent with the markedly higher docking scores observed for TLR2 and PAR2 relative to the other candidates.

The nonapeptide also displayed moderate predicted affinities towards EGFR and TGFβR. Within the predicted EGFR complex, the peptide forms hydrogen bonds with Arg841, Asn842, Ser720, Leu718, Lys716, and Glu804, further stabilized by salt bridges at Asp855 and Glu865. For TGFβR, hydrogen bonds are primarily predicted with Gly214, Gly217, Arg215, and Asp435, supported by an attractive charge interaction at Glu238. However, compared to TLR2/PAR2, the overall interaction networks for EGFR and TGFβR are less extensive.

Consistent with their lower predicted binding scores, the modeled interactions between GPSGPQGSR and the structural tight junction proteins are relatively limited in both depth and density. Although hydrogen bonds are predicted between the peptide and residues of Occludin (Tyr481, Asp461, Leu464, Tyr474, Lys485, Lys488) and Claudin-1 (Asp108, Tyr111, Gly194, Lys191), the depth of pocket insertion and the density of the surrounding hydrophobic and van der Waals contacts are noticeably inferior to those of the top-ranked receptor complexes. Collectively, these computational findings identify TLR2 and PAR2 as the most promising candidate receptors for GPSGPQGSR among the ten targets screened and provide a structural basis for prioritizing these two receptors in subsequent experimental validation.

### 2.4. Molecular Dynamics Simulations of GPSGPQGSR–Receptor Complex

To assess the favorable static docking poses predicted for the top-ranked complex, which would remain stable under dynamic physiological conditions, 100 ns molecular dynamics (MD) simulations were conducted for the six highest-ranked peptide–receptor complexes. Structural stability, compactness, residue-level flexibility, and intermolecular hydrogen bonding were evaluated using Root Mean Square Deviation (RMSD), Radius of Gyration (Rg), Solvent Accessible Surface Area (SASA), Root Mean Square Fluctuation (RMSF), and hydrogen bond number metrics.

**Figure 5:**
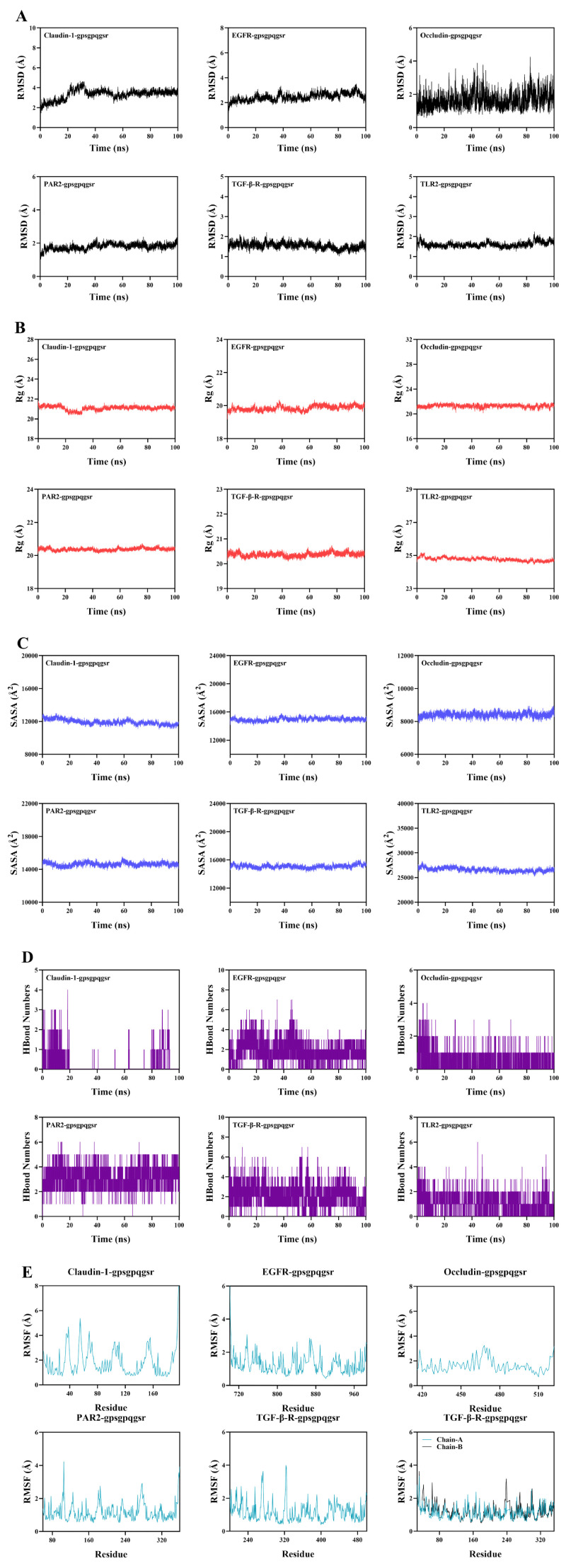
Molecular dynamics simulation of the protein–ligand complex. (**A**), Time-dependent RMSD values of the protein–ligand complex; (**B**), Time-dependent Rg values of the protein–ligand complex; (**C**), Time-dependent SASA values of the protein–ligand complex; (**D**), Time-dependent number of hydrogen bonds (H-Bonds) in the protein–ligand complex; (**E**), RMSF values of the protein–ligand complex.

The RMSD of the protein backbone serves as a primary indicator of overall structural stability throughout the simulation trajectory with values consistently below 3.0 Å [40]. Figure 5A shows that the TLR2-GPSGPQGSR and PAR2-GPSGPQGSR complexes demonstrated markedly stable trajectories, with TLR2 converging to approximately 1.8 Å within the first 5 ns and PAR2 reaching equilibrium at approximately 2.2 Å by 35 ns. The TGFβR complex similarly maintained high stability throughout the simulation at about 1.6 Å. Conversely, the Claudin-1 complex exhibited a substantially less stable trajectory, taking about 55 ns to reach a plateau at a higher RMSD of approximately 3.8 Å, suggesting that the peptide-Claudin-1 binding mode is considerably less stable in a dynamic environment than the top-ranked complexes.

The Rg and SASA profiles (Figure 5B,C) for TLR2, PAR2, Occludin, and TGFβR complexes remained relatively stable throughout the simulation, indicating that peptide binding did not lead to gross unfolding or abnormal compaction of the receptor architecture in these complexes. The EGFR complex, despite its moderate docking score, exhibited slightly greater fluctuations in both Rg and SASA compared to TLR2 and PAR2. This apparent discrepancy between EGFR’s intermediate docking score and its relatively greater dynamic instability is noteworthy: while static docking scores primarily reflect the enthalpic contribution of predicted contacts at a fixed pose, MD simulations additionally capture entropic effects, solvation dynamics, and conformational breathing of the receptor, all of which can substantially influence the stability of a bound complex. The greater conformational flexibility of the EGFR kinase domain relative to the more rigid extracellular binding pocket of TLR2 may therefore account for this divergence.

The RMSF profiles (Figure 5E) revealed that residue-level atomic fluctuations in the PAR2 and TLR2 binding complexes were predominantly confined to below 3.0 Å, as opposed to less than 5.0 Å for Claudin-1. According to principles of structural pharmacology, such reduced regional flexibility at the binding interface implies that the peptide ligand effectively constrains the conformational dynamic of the active pocket, thereby stabilizing the receptor in a bonding conformation [41].

The divergence in structural stability across complexes is closely mirrored by the intermolecular hydrogen bond dynamics (Figure 5D). During the 100 ns simulation, the nonapeptide maintained the most persistent hydrogen bond networks with PAR2 (frequently maintaining 4 bonds, with a maximum of 6) and TLR2 (frequently maintaining 3 bonds, with a maximum of 6). These sustained hydrogen bond networks are consistent with the high docking scores predicted for these two receptors and support their designation as primary predicted binding partners of GPSGPQGSR. In contrast, the Claudin-1 complex sustained on average only about 1 intermolecular hydrogen bond through the simulation, suggesting a transient and superficial interaction susceptible to competitive displacement by solvent molecules.

Taken together, both the static molecular docking and the 100 ns MD simulations offer convergent computational evidence that GPSGPQGSR forms stable, specific predicted interactions preferentially with TLR2 and PAR2 over the other candidate proteins examined. These simulation results strengthen the prioritization of TLR2 and PAR2 as the most plausible extracellular targets of the nonapeptide. It should be emphasized, however, that these findings remain computational predictions, and definitive conclusions regarding the in vitro binding partners and mechanism of action of GPSGPQGSR require experimental corroboration.

### 2.5. Effects of TLR2 and PAR2 Blockade on the Barrier-Protective Function of GPSGPQGSR

To verify the computational predictions arising from molecular docking and MD simulations, pharmacological receptor blockade experiments were conducted using a TNFα-challenged Caco-2 cell model. The PAR2-selective antagonist FSLLRY-NH_2_ and the TLR2-specific inhibitor C29 were employed. Barrier integrity was assessed using TEER and FD4 permeability. Cell viability under all experimental conditions was confirmed to be unaffected by MTT assay.

**Figure 6:**
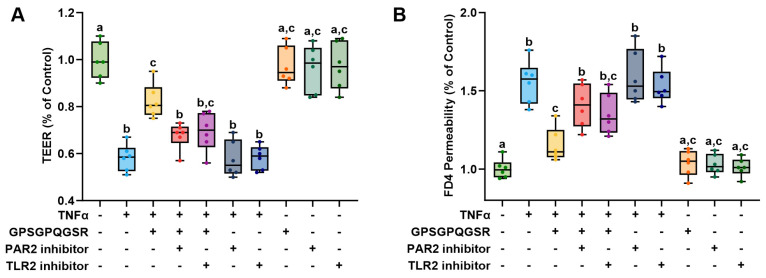
Effects of TLR2 and PAR2 inhibitors on the barrier-protective activity of GPSGPQGSR. (**A**) TEER values expressed as a percentage of the normal control. (**B**) FD4 flux reflecting paracellular permeability, expressed as fold change relative to control. Data are mean ± SD (*n* = 6). Groups sharing the same superscript letter are not significantly different (Tukey–Kramer HSD, *p* < 0.05).

As illustrated in Figure 6A, administration of GPSGPQGSR, the PAR2 antagonist FSLLRY-NH_2_, or the TLR2 inhibitor C29 alone to intact monolayers (without TNFα challenge) did not significantly alter TEER values compared to the untreated control group. This indicates that neither the peptide nor the inhibitors independently perturb baseline barrier function. Upon TNFα challenge, monolayer barrier integrity was substantially compromised, as evidenced by an approximately 40% reduction in TEER relative to the control group. Co-incubation with GPSGPQGSR significantly ameliorated the TNFα-induced barrier dysfunction, restoring TEER to approximately 85% of the control level. Notably, treatment with either inhibitor alone under TNFα challenge (in the absence of GPSGPQGSR) resulted in TEER values indistinguishable from the TNFα model group, confirming that the inhibitors do not independently exacerbate inflammatory barrier damage. Critically, pre-treatment with either the PAR2 antagonist FSLLRY-NH_2_ or the TLR2 inhibitor C29 completely abolished the barrier protective effect of GPSGPQGSR, with TEER values in both inhibitor-plus-peptide groups reverting to levels comparable to the TNFα model group alone.

This pattern of response was consistently observed in the paracellular permeability assay (Figure 6B). TNFα stimulation induced a substantial approximately 1.6-fold increase in FD4 flux relative to the untreated control, while GPSGPQGSR or inhibitors alone did not significantly alter baseline FD4 permeability. Treatment with GPSGPQGSR effectively reduced TNFα-induced FD4 leakage. In contrast, pre-treatment with either PAR2 or TLR2 inhibitors fully negated this protective effect. Because the inhibitors alone did not worsen FD4 leakage under TNFα stimulation, the loss of protection in the blockade groups can be exclusively attributed to the antagonism of the target receptors. These robust pharmacological findings unambiguously demonstrated that the barrier-protective function of GPSGPQGSR operates in a receptor-dependent manner, requiring functional TLR2 and PAR2 signalling. The downstream signalling mechanisms through which TLR2 and PAR2 engagement may restore barrier integrity warrant detailed consideration. Upon activation by specific ligands, apically localized TLR2 recruits the adaptor protein MyD88, initiating a signalling cascade that activates PI3K and its downstream effector Akt. Phosphorylated Akt has been shown to directly phosphorylate and inactivate glycogen synthase kinase-3β (GSK-3β), thereby relieving its inhibitory effect on tight junction assembly and promoting the membrane localization of ZO-1 and Occludin [39,42]. In parallel, TLR2-mediated PKC activation, particularly the PKCδ isoform, has been reported to enhance the phosphorylation-dependent recruitment of Claudin-1 and ZO-1 to apical junctional complexes, reinforcing paracellular sealing under inflammatory conditions [42,43]. Regarding PAR2, its functional outcome is critically determined by the mode of activation. Whereas proteolytic cleavage of the PAR2 N-terminal domain by serine proteases drives Gαq-mediated calcium signalling and NF-κB activation that disrupts tight junction architecture, activation by specific synthetic or endogenous peptide ligands has been reported to preferentially engage β-arrestin-dependent, G protein-independent pathways [44]. This biased signalling stabilizes the internalized receptor–β-arrestin scaffold, which serves as a platform for ERK1/2 activation in endosomal compartments, promoting epithelial restitution and mucosal repair without triggering the pro-inflammatory Gαq cascade [44,45]. It is therefore plausible that GPSGPQGSR, as a peptide ligand, may function as a biased PAR2 agonist while simultaneously engaging TLR2-mediated pro-barrier signalling, thereby activating complementary pathways that cooperatively converge on tight junction stabilization. Potential cross-talk between TLR2 and PAR2 cascades—for example, through shared downstream regulation of ZO-1 phosphorylation status or NF-κB suppression—represents an important mechanistic question that remains to be addressed in future studies.

**Figure 7:**
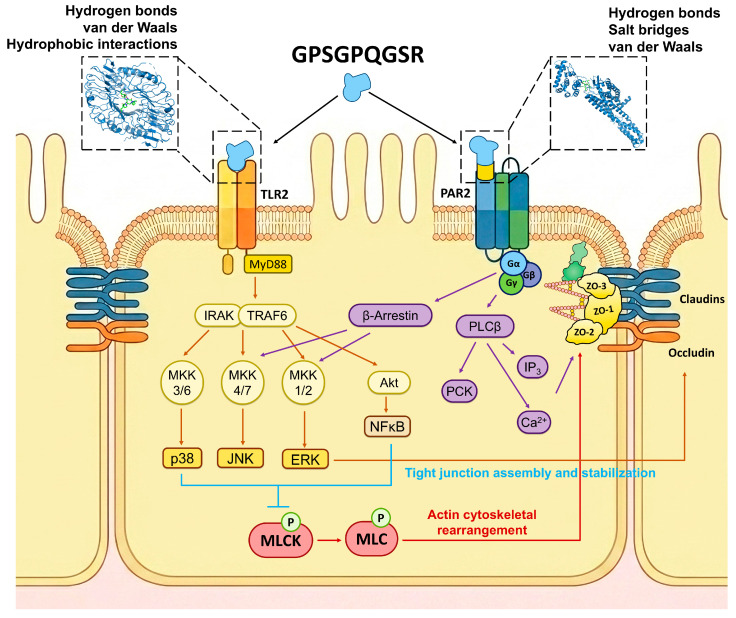
Proposed hypothetical model of GPSGPQGSR in intestinal-barrier protection.

Figure 7 presents a proposed mechanistic model synthesizing the above evidence. This hypothetical framework positions GPSGPQGSR as an apically retained luminal ligand that engages TLR2 and PAR2 on the enterocyte surface, initiating convergent signalling programs directed toward tight junction assembly and barrier restoration. This model is explicitly a working hypothesis rather than a fully validated pathway; the precise binding interfaces, downstream signalling intermediates, relative contributions of each receptor, and potential TLR2–PAR2 cross-talk all remain to be confirmed through targeted biochemical and cell biological investigation.

The study indicates that GPSGPQGSR has potential for treating conditions linked to intestinal barrier dysfunction, such as IBD, IBS with increased permeability, chemotherapy-induced mucositis, and non-alcoholic fatty liver disease. This peptide acts directly on the epithelial surface, reducing systemic exposure and off-target effects compared to traditional anti-inflammatory drugs. Its collagen-derived nature and resistance to digestion make it suitable for oral delivery, suggesting its use in functional foods and nutraceuticals like fermented dairy, collagen supplements, or encapsulated products.

### 2.6. Technical Challenges and Future Research Directions

Despite promising in vitro evidence, several challenges must be addressed before GPSGPQGSR can be used clinically or commercially. Validation in animal models of intestinal barrier dysfunction is needed to confirm in vivo efficacy, as the Caco-2 model is insufficient. The peptide’s stability in the colonic environment is unknown, so encapsulation strategies might be necessary. Its stability during food processing also needs evaluation for potential food-based delivery. Production methods, such as enzymatic hydrolysis or recombinant systems, require optimization for scalability and cost-effectiveness. Safety profiling, including assessments of mucosal immunogenicity and allergenicity, is essential before human use. Additionally, it is crucial to systematically explore the TLR2–PAR2 signalling pathways and receptor interactions using phosphoproteomic and organoid methods. Addressing these challenges systematically will be essential to realize the therapeutic and nutraceutical potential of GPSGPQGSR.

## 3. Materials and Methods

### 3.1. Materials and Reagents

The nonapeptide GPSGPQGSR (>99% purity) was chemically synthesized by QiangYao Biotechnology Co., Ltd. (Shanghai, China). The Caco-2 human colorectal adenocarcinoma cell line was acquired from the Cell Bank of the Chinese Academy of Sciences (Shanghai, China). Dulbecco’s Modified Eagle’s Medium (DMEM, high glucose), fetal bovine serum (FBS), penicillin-streptomycin solution (10,000 U/mL), Hank’s Balanced Salt Solution (HBSS), and trypsin-EDTA (0.25%) were supplied by Thermo Fisher Scientific (Gibco™, Grand Island, NY, USA). Pepsin (P7012), pancreatin (P7545), recombinant human tumor necrosis factor-α (TNFα, T6674), fluorescein isothiocyanate-dextran (FD4), Gly-Sar, Cytochalasin D, Wortmannin, FSLLRY-NH_2_ and C29 were bought from Sigma-Aldrich (Shanghai, China). Other chemicals were of analytical or LC-MS grade.

### 3.2. In Vitro Simulated Gastrointestinal Digestion

To evaluate the resistance of GPSGPQGSR to gastrointestinal proteases, a simplified static digestion assay adapted from the INFOGEST consensus protocol was employed [46]. The oral phase was omitted, as the primary aim of this study was to assess peptide resistance specifically to gastric and intestinal proteolytic enzymes rather than to simulate the complete digestion of a food matrix.

Gastric digestion phase: The peptide GPSGPQGSR (1 mg/mL) was mixed with simulated gastric fluid (SGF) containing pepsin (25,000 U/mL) at pH 2.0. The enzyme-to-substrate ratio used was 10:1 (*w*/*w*). The mixture was incubated at 37 °C with continuous shaking at 100 rpm for 2 h.

Intestinal digestion phase: Following gastric digestion, the pH of the mixture was adjusted to 7.0 using 1 M NaHCO_3_. Simulated intestinal fluid (SIF) containing pancreatin (800 U/mL) was added. The enzyme-to-substrate ratio was 4:1 (*w*/*w*). Bile salts were deliberately excluded from the SIF, as GPSGPQGSR is a pure peptide substrate rather than a lipid-containing food matrix, and the primary objective was to evaluate susceptibility to pancreatic proteases. The mixture was subsequently incubated at 37 °C with continuous shaking at 100 rpm for 2.5 h.

Enzyme inactivation and sample preparation: Enzymatic reactions were terminated by heating at 100 °C for 5 min. Samples were subsequently centrifuged at 5000× *g* for 5 min, 4 °C, and the supernatants were collected, desalted using C18 solid-phase extraction cartridges, lyophilized, and stored at −80 °C prior to UPLC-Q-TOF-MS/MS analysis.

Experimental controls: three control groups were included in parallel (i) an enzyme blank (pepsin or pancreatin incubated under identical conditions without peptide substrate), to account for potential enzyme autolysis signals; (ii) a substrate blank (peptide incubated under identical pH and temperature conditions without enzymes), to assess non-enzymatic degradation; and (iii) an undigested peptide control (S0), representing the peptide at time zero before any digestion treatment.

All digestion experiments were performed in three independent biological replicates (*n* = 3), each conducted on separate days with freshly prepared enzyme solutions, to ensure reproducibility.

### 3.3. Identification and Quantification of Peptides via UPLC-Q-TOF-MS/MS

To accurately identify the intact GPSGPQGSR peptide and its potential metabolites (degradation fragments) produced during simulated digestion and transepithelial transport, an Agilent 1290 Infinity UPLC system, coupled with an Agilent G6540 Quadrupole Time-of-Flight (Q-TOF) mass spectrometer (Agilent Technologies Inc., Santa Clara, CA, USA), was utilized [47,48].

Chromatographic conditions: Samples (10 μL) were filtered through a 0.22 μm PVDF membrane prior to injection. Chromatographic separation was performed on an Agilent Advance Bio Peptide Plus C_18_ column (150 mm × 2.1 mm, 130 Å, 2.7 μm, Agilent Technologies Inc., Santa Clara, CA, USA) at a column temperature of 30 °C. The mobile phase consisted of (A) 0.1% formic acid in water (*v*/*v*) and (B) acetonitrile containing 0.1% formic acid (*v*/*v*). The flow rate was set at 0.2 mL/min. A linear gradient elution program was applied as follows: 0–50 min, 10–90% B; 50–55 min, 90% B; 55–56 min, 90–10% B; 56–60 min, 10% B (re-equilibration).

Mass spectrometry conditions: The Q-TOF-MS was operated in positive electrospray ionization (ESI+) mode with a data-dependent acquisition (DDA) strategy. Key instrumental parameters were as follows: drying gas temperature, 280 °C; drying gas flow rate, 5 L/min; sheath gas temperature, 280 °C, sheath gas flow rate, 11 L/min; nebulizer pressure, 2.1 × 10^5^ Pa; capillary voltage, 3.5 kV; nozzle voltage, 500 V; fragmentor voltage, 135 V. Full MS scans were acquired at a mass resolution of 35,000 FWHM, and data-dependent MS/MS scans were acquired at 17,500 FWHM with collision energies of 25 eV and 50 eV applied for fragmentation.

Quantification and method validation: For quantitative analysis of intact GPSGPQGSR, an external calibration curve was constructed using a serial dilution of synthetic GPSGPQGSR standard solutions at concentrations of 0.01, 0.05, 0.1, 0.5, 1.0, 2.0, 5.0 μg/mL. The calibration curve exhibited linearity over this range with a coefficient of determination (R^2^) of ≥0.99. The limit of detection (LOD) and limit of quantification (LOQ) were determined based on a signal-to-noise ratio of 3 and 10, respectively. It should be noted that ionization efficiency may differ between the intact nonapeptide and its degradation fragments due to differences in molecular weight, charge state, and amino acid composition. Therefore, quantitative comparisons between intact GPSGPQGSR and identified metabolites are semi-quantitative in nature and are primarily used to assess relative abundance changes.

Data processing: Raw mass spectrometry data were processed using MaxQuant (v1.5.0.12). Variable modifications included N-terminal acetylation and methionine oxidation. Pepsin and trypsin were specified as the proteolytic enzymes, allowing a maximum of two missed cleavages. The peptide-level false discovery rate (FDR) was set at 1%, with a minimum peptide length of six amino acids.

### 3.4. Transepithelial Transport Experiment

Cell culture and monolayer preparation: Caco-2 cells (passage 20–30) were seeded onto polycarbonate Transwell insert plates with a pore size of 0.4 μm (Corning Inc., Corning, NY, USA) at a density of 1 × 10^5^ cells/cm^2^. Cells were maintained in DMEM supplemented with 10% (*v*/*v*) FBS and 1% (*v*/*v*) penicillin-streptomycin at 37 °C in a humidified atmosphere containing 5% CO_2_. Culture medium was replaced every 2 days. Cells were cultured for 21 days to allow complete differentiation and formation of confluent monolayers with mature tight junctions, as previously described [19].

Monolayer integrity assessment: The transepithelial electrical resistance (TEER) was continuously measured using a Millipore ERS-2 volt-ohm meter (Millipore, Burlington, MA, USA) throughout the 21-day culture period. Immediately before and after each 2 h transport experiment, TEER values were recorded to confirm monolayer integrity. Only monolayers with pre-experiment TEER values exceeding 400 Ω·cm^2^ were selected for use. Monolayers in which post-experiment TEER values declined by more than 15% relative to the pre-experiment baseline were excluded from analysis [49]. Additionally, paracellular macromolecular permeability was validated using FD4 under the same transport conditions. All monolayers meeting the TEER criterion (>400 Ω·cm^2^) exhibited FD4 apparent permeability coefficients (Papp) below 1.0 × 10^−6^ cm/s.

Transport experiment and sample collection: To investigate the spatial distribution and apical retention of the peptide, the monolayers were rinsed with warm phosphate-buffered saline (PBS). Subsequently, 0.5 mL of GPSGPQGSR peptide solution (1 mg/mL in Hank’s Balanced Salt Solution, HBSS) was added to the apical (AP) chamber, while 1.5 mL of HBSS was added to the basolateral (BL) chamber. After a 2 h incubation at 37 °C in a 5% CO_2_ atmosphere, samples were collected separately from the AP chamber, BL chamber, and intracellular lysates. These samples were then lyophilized and analyzed using UPLC-Q-TOF-MS/MS, as detailed in Section 3.3, to determine the precise distribution and fate of the peptide and its metabolites.

### 3.5. Molecular Docking Screening of Putative Membrane Targets

Molecular docking was utilized to systematically investigate the potential extracellular targets of GPSGPQGSR, motivated by its notable apical retention profile. Ten critical transmembrane receptors, selected based on previous studies, associated with the intestinal barrier and immune response were identified: Claudin-1, Claudin-2, Occludin, Epidermal Growth Factor Receptor (EGFR), Protease-Activated Receptor 2 (PAR2), Transforming Growth Factor-Beta Receptor (TGFβR), Toll-Like Receptor 2 (TLR2), Vascular Endothelial Growth Factor Receptor (VEGFR), Calcium-Sensing Receptor (CaSR), and C-X-C Chemokine Receptor Type 1 (CXCR1). The three-dimensional crystal structures of these receptors were obtained from the RCSB Protein Data Bank (PDB), except for Claudin-1, which was constructed via homology modeling using MOE (v2014.09011) software, based on the PDB template 3X29. Prior to docking, the target proteins were protonated and structurally optimized using the Structure Preparation module in MOE, employing the AMBER10: EHT force field and R-field implicit solvent model. Flexible induced-fit docking was conducted to calculate the binding affinities, facilitating the identification of the most promising candidate receptors for subsequent dynamic validation.

### 3.6. Molecular Dynamics Simulations (MDSs)

To validate the dynamic stability of peptide–receptor complexes from molecular docking, 100 ns MDS were performed using GROMACS (v2022) [50]. The AMBER14SB force field was applied to receptor proteins, while GAFF2 force field files for the GPSGPQGSR peptide were generated with sobtop (v1.0), using RESP for atomic charges. Complexes were solvated in a 1 nm cubic box with TIP3P water, and Na^+^/Cl^−^ ions were added for 0.15 M NaCl concentration. The Particle Mesh Ewald method managed electrostatic interactions, and LINCS constrained covalent bonds. Energy minimization was done in three stages: minimizing water with solute constraints, minimizing with ion constraints, and global minimization. Simulations were conducted using the NPT ensemble at 310 K and 1 bar, controlled by the Nosé–Hoover thermostat and Parrinello–Rahman barostat, respectively. The molecular dynamics ran for 100 ns with a 2-fs time step. Structural trajectories were analyzed with GROMACS to determine RMSD, RMSF, Rg, SASA, and intermolecular hydrogen bonds.

### 3.7. Receptor-Dependent In Vitro Validation

To validate computational predictions about PAR2 and TLR2, a TNFα-induced inflammatory Caco-2 monolayer model was used. Mature Caco-2 monolayers (TEER > 400 Ω·cm^2^) were pre-incubated with inhibitors FSLLRY-NH_2_ (100 μM for PAR2) or C29 (50 μM for TLR2) on the apical side for 1 h. The peptide GPSGPQGSR (1 mg/mL) was then added and incubated for 24 h. After this, the basolateral medium was replaced with 10 ng/mL TNFα to induce inflammation. After 24 h, barrier integrity was assessed by measuring TEER and paracellular permeability using 4 kDa FITC-dextran. If the inhibitors significantly reduced the peptide’s protective effects, it would confirm the mechanism of barrier fortification.

### 3.8. Statistical Analysis

The experiments were performed in a minimum of three independent replicates. Data are presented as the mean ± standard deviation (SD). Statistical significance was evaluated using one-way analysis of variance (ANOVA), followed by the Tukey–Kramer multiple comparison post hoc test, employing GraphPad Prism (v10.1.2). A *p*-value of less than 0.05 was deemed statistically significant.

## 4. Conclusions

This study comprehensively elucidates the gastrointestinal fate and receptor-mediated intestinal barrier-protective mechanisms of the Alaska pollock skin-derived collagen peptide GPSGPQGSR. The results demonstrate that GPSGPQGSR exhibits exceptional resistance to gastrointestinal proteolysis, enabling it to reach the intestinal epithelium as an intact nonapeptide. Despite partial degradation by brush border enzymes, up to 42.16% of the intact peptide is specifically retained on the luminal surface, with negligible cellular internalization and basolateral transepithelial translocation. Supported by molecular docking, molecular dynamics simulations, and in vitro pharmacological blockade assays, we confirmed that the luminally retained GPSGPQGSR acts as a high-affinity extracellular ligand. It binds to the surface receptors TLR2 and PAR2, actively triggering their downstream protective signalling cascades to effectively restore and maintain tight junction barrier function.

These findings successfully decipher the scientific paradox between the low systemic bioavailability and high physiological bioactivity of bioactive peptides, establishing a novel paradigm in which peptides exert targeted efficacy as extracellular signalling ligands on the intestinal luminal side. Ultimately, this research provides a theoretical foundation and an innovative dimension for the precise translation of marine-derived bioactive peptides into functional ingredients or oral targeted biotherapeutics for the management of intestinal barrier function.

## Figures and Tables

**Table 1:** The docking score of the peptide binding to ten targets.

Targets	Binding Energy Score (Kcal/mol)	Targets	Binding Energy Score (Kcal/mol)
TLR2	−14.936	Claudin-1	−6.513
PAR2	−10.154	Claudin-2	−5.238
TGFβR	−7.714	VEGFR	−5.052
EGFR	−7.615	CaSR	−4.788
Occludin	−6.621	CXCR1	−4.566

## Data Availability

The authors declare that the supporting data of this study are available within the article.
